# The Immediate Carryover Effects of Peroneal Functional Electrical Stimulation Differ between People with and without Chronic Ankle Instability

**DOI:** 10.3390/s22041622

**Published:** 2022-02-18

**Authors:** Uri Gottlieb, Jay R. Hoffman, Shmuel Springer

**Affiliations:** Neuromuscular and Human Performance Laboratory, Department of Physiotherapy, Faculty of Health Sciences, Ariel University, Ariel 40700, Israel; urig@ariel.ac.il (U.G.); jayho@ariel.ac.il (J.R.H.)

**Keywords:** chronic ankle instability, functional electrical stimulation, statistical parametric mapping, kinematics, gait analysis

## Abstract

Chronic ankle instability (CAI) is a common condition that may develop after an ankle sprain. Compared with healthy individuals, those with CAI demonstrate excessive ankle inversion and increased peroneal electromyography (EMG) activity throughout the stance phase of gait, which may put them at greater risk for re-injury. Functional electrical stimulation (FES) of targeted muscles may provide benefits as a treatment modality to stimulate immediate adaptation of the neuromuscular system. The present study investigated the effect of a single, 10 min peroneal FES session on ankle kinematics and peroneal EMG activity in individuals with (*n* = 24) or without (*n* = 24) CAI. There were no significant differences in ankle kinematics between the groups before the intervention. However, after the intervention, healthy controls demonstrated significantly less ankle inversion between 0–9% (*p* = 0.009) and 82–87% (*p* = 0.011) of the stance phase. Furthermore, a significant within-group difference was observed only in the control group, demonstrating increased ankle eversion between 0–7% (*p* = 0.011) and 67–81% (*p* = 0.006) of the stance phase after the intervention. Peroneal EMG activity did not differ between groups or measurements. These findings, which demonstrate that peroneal FES can induce ankle kinematics adaptations during gait, can help to develop future interventions for people with CAI.

## 1. Introduction

Chronic ankle instability (CAI) is a condition characterized by repetitive episodes of the perception that the ankle is unable to support continued locomotion. This may be accompanied by pain, local muscle weakness, and/or reduced range of motion in the ankle joint. CAI is diagnosed when recurrent ankle sprains persist for more than 1 year after the initial injury [1], and it may develop in up to 40–60% of patients with a history of an ankle sprain [2,3]. CAI has a negative effect on people’s lives, both in the short and long term. Decreased quality of life [4,5,6,7,8] and reduced physical activity [9,10,11,12] were reported in patients with CAI. Furthermore, earlier onset of ankle joint osteoarthritis is linked to the presence of this condition [13,14,15,16,17,18,19].

When compared with healthy subjects, individuals with CAI display sensorimotor control deficits such as reduced postural stability [20,21], reduced peroneal muscles strength [22], and altered kinematics and muscle activation patterns during gait [23]. Specifically, people with CAI demonstrate an excessive ankle and foot inversion angle [24,25,26,27] and increased peroneal muscle activation [28,29,30] during the stance phase of gait. These gait impairments may contribute to a lateral deviation of the center of pressure [30] and may increase the likelihood for ankle re-sprain. Increased peroneal activation after heel strike is presumed to be a representation of a feed-forward mechanism that people with CAI use to avoid the inverted ankle position [28]. Nevertheless, it seems that the elevated muscle activation is not successful in preventing increased ankle inversion, which may imply a muscular activation failure. A factor that is associated with altered motor control in this population is atherogenic muscle inhibition (AMI)—an ongoing reflex inhibition of muscles surrounding an injured joint [31]. Evidence for reduced spinal and possibly cortical excitability of the peroneus longus muscle in individuals with CAI has been previously reported and summarized [32].

Neuromuscular electrical stimulation (NMES) is a potential therapeutic agent to overcome AMI [33]. When applying electrical stimuli over the innervating nerve, it can produce muscle contraction, bypassing spinal or supraspinal inhibition. The sensory input produced by electrical stimulation (ES) has been shown to induce cortical excitability changes in both the motor cortex [34,35,36] and spinal cord [37,38] and is associated with the reorganization of cortical motor maps of the corresponding muscle. When NMES is delivered in coordination with a functional task, such as walking, it is referred to as functional electrical stimulation (FES). FES may result in orthotic, immediate carryover, or therapeutic effects [39]. An orthotic effect is defined as an improvement observed while using FES, compared with baseline. The immediate carryover effect describes the short-term consequences after applying FES. A therapeutic effect, sometimes defined as the training effect, refers to the long-term outcomes of FES training—namely, performing a functional task without FES support.

FES provides coordinated, rhythmic stimulation, in a pattern that imitates normal movement and therefore contributes to the recovery of muscle control [40]. Furthermore, it may enhance activation of target muscles resulting in immediate adaptation of the neural system and enhanced neuromuscular control [41]. In addition, earlier studies have shown that FES may facilitate spinal [42] and cortical motor excitability [43,44], especially when combined with voluntary repeated movements. These mechanisms may explain immediate or long-lasting gait modifications following FES observed in patients with neurological conditions [45,46,47]. For example, it has been shown that gait speed and cadence improved following FES of the dorsiflexors in patients post-stroke [45]. These effects may be of importance since preliminary evidence suggests a link between immediate carryover effects and long-term therapeutic effects [46,48]. It should be noted that most of the research investigating the effects of FES has focused on evaluating the orthotic and therapeutic effects, meaning that data were collected during FES application or after a series of training sessions. Immediate carryover effects of FES have been far less reported in the literature.

It has been previously shown that FES provided in concurrence with gait training is more beneficial in restoring muscles strength and control after anterior cruciate ligament reconstruction when compared with NMES [49]. Furthermore, recent evidence suggests that a single peroneal FES session had a positive, acute carryover effect on standing balance assessment in a sample of young, healthy females [50]. Therefore, it is reasonable to presume that FES might improve motor control of the peroneal muscles in people with CAI.

The objective of this study was to investigate the immediate carryover effects of a single gait training with peroneal FES on ankle kinematics and peroneal activity in individuals with and without CAI. Completing these objectives should convey primary information for future interventional studies that will explore the potential benefits of FES in CAI rehabilitation. Furthermore, comparing FES effects between these groups may expand the understanding of the impaired gait in CAI. We hypothesized that applying peroneal FES during 0–80% of the stance phase of gait will decrease ankle inversion angle after heel contact and decrease muscle activation in both groups (i.e., normalize gait patterns in the CAI group).

## 2. Materials and Methods

### 2.1. Participants

The sample size for this study was determined based on a power analysis simulation, using the Power1D package for Python [51]. We used gait data of healthy, young adults that were previously collected as baseline data. Then, we simulated CAI data, assuming a 3° increased inversion angle during the initial 10% of the gait cycle, and an intersubject standard deviation of 4°. Using these simulated data, a sample size of 24 subjects per group was required to reach a power >0.8. Consequently, 24 subjects with CAI and 24 healthy controls participated in the study. The enrollment criteria for the CAI group were set according to the position statement of the International Ankle Consortium for controlled studies [52]. These criteria included (1) history of at least one significant ankle sprain that occurred at least 12 months before the study and was diagnosed by a physician or a physical therapist based on clinical examination [53]; (2) history of at least two episodes of “giving way” and feelings of ankle joint instability, at least twice during the previous 6 months; (3) the most recent injury occurred more than 3 months before study enrollment; (4) scoring ≤ 24 in the Cumberland Ankle Instability Tool (CAIT) [54]; (5) able to bear full weight on the injured lower extremity with no more than mild discomfort. The control group included age-matched healthy participants with no complaints about lower extremity pain or dysfunction. Healthy subjects were not included if they had sustained an ankle sprain in the previous 12 months before enrollment.

Exclusion criteria for all groups were a history of lower extremity fracture that required realignment, surgical procedures, or other pathological conditions in the lower extremities, and vestibular or neurological disorders. Participants were recruited by advertising the study in the university facilities and social media. All participants provided written informed consent before participating in the study. Ariel University Institutional Review Board approved the study (Approval Number: AU-HEA-SS-20200308). The study was pre-registered at ClinicalTrials.gov (Identifier: NCT04314960).

### 2.2. Procedure

The study was conducted during a single session at the Neuromuscular and Human Performance Laboratory, at Ariel University. Demographic variables (age, stature, body weight, and leg dominancy) were collected from all participants. Additionally, CAIT scores were collected for each limb separately, as well as the Foot and Ankle Ability Measure (FAAM) [55]. CAIT is a 9-item, 30-point scale for measuring the severity of functional ankle instability, with a higher score indicating better condition [54]. The FAAM assessment is a self-reported functional outcome measure utilized to track subjective changes in perceived pain and ankle function. It consists of 21 items in two subscales—ADL and sports—each ranging between 0 and 100, with a higher score indicating better functionality [55].

Ankle kinematics and peroneal activity were assessed during walking on a motorized treadmill (VO_2_ Challenger, Taipei, Taiwan) for 30 s before and immediately after a 10 min peroneal FES. To synchronize kinematic and muscle activation data collection, an external trigger was used to start both recordings simultaneously. Participants walked barefoot and wore tight, black sports pants and t-shirts. To determine gait speed, the treadmill was set at 3 km/h (0.83 m/s). Then, participants were instructed to increase the speed (using a remote control and without seeing the treadmill screen) until reaching a comfortable, self-selected pace (SSP). The instruction given was “walk at the speed you would walk in the street if you were slightly in a hurry”. After determining SSP, walking speed was increased by 20% (SSP + 20%) for gait measurement, since it was previously reported that people with CAI demonstrate greater ankle inversion as speed increases [56]. Before data collection, participants were allowed to habituate to walking on the treadmill.

### 2.3. Ankle Kinematics

To capture ankle kinematics, reflective markers were placed directly on the skin using double-sided tape. A total of 13 reflective markers were placed on each side of the participants’ anterior and posterior superior iliac spines, greater trochanter, lateral and medial femoral condyles, ankle medial and lateral malleoli, heel, first and fifth metatarsal’s heads and bases, and between the bases of the second and third metatarsal. In addition, cluster markers were placed at mid-thigh and mid-calf. An eight-camera motion capture system (Qualisys, Göteborg, Sweden) sampled at 100 Hz was used to obtain three-dimensional ankle kinematics. Data were exported to Visual 3D software (C-motion, Inc., Kingston, ON, Canada), and processed through a 6 degree-of-freedom anthropometric model. Ankle angles in the frontal plane during walking were calculated using the Cardan rotation sequence [57]. The raw angle data were then filtered using a first-order, low-pass, 10 Hz Butterworth filter and smoothened by a third-order Savitzky–Golay filter [58]. Each measurement was then divided into separate stance phases (from heel contact to the same foot next toe-off). Heel contacts and toe-offs were identified as the time point when the displacement of the foot with relation to the pelvis reached its maximum or minimum distance in the walking direction, respectively [59]. The ankle frontal plane angle throughout the stance phase was transformed to 101 data points for each gait cycle and averaged for each data point.

### 2.4. Peroneal Activity

Peroneal electromyography (EMG) activity was collected using a wireless EMG system (Delsys Trigno, Delsys Inc., Boston, MA, USA) at 2000 Hz. Before data collection, participants’ skin was shaved and cleaned with alcohol to minimize impedance. Electrode placement was performed according to Surface Electromyography for the Noninvasive Assessment of Muscles (SENIAM) guidelines [60]. Before walking, EMG activity during maximal voluntary isometric contraction (MVIC) was measured against manual resistance. Each MVIC was performed three times for 3 s, with 60 s rest between contractions. The raw EMG signal was digitally filtered to remove baseline noise (20 Hz/400 Hz, fourth-order, band-pass Butterworth filter), rectified with a moving root mean square (window size 0.125 ms), and normalized to %MVIC to establish a common ground to compare data between participants. The normalized EMG amplitude during the stance phase was handled in a similar approach as the kinematic data (i.e., transformed to 101 data points and averaged per subject).

### 2.5. Peroneal FES

**Figure 1:**
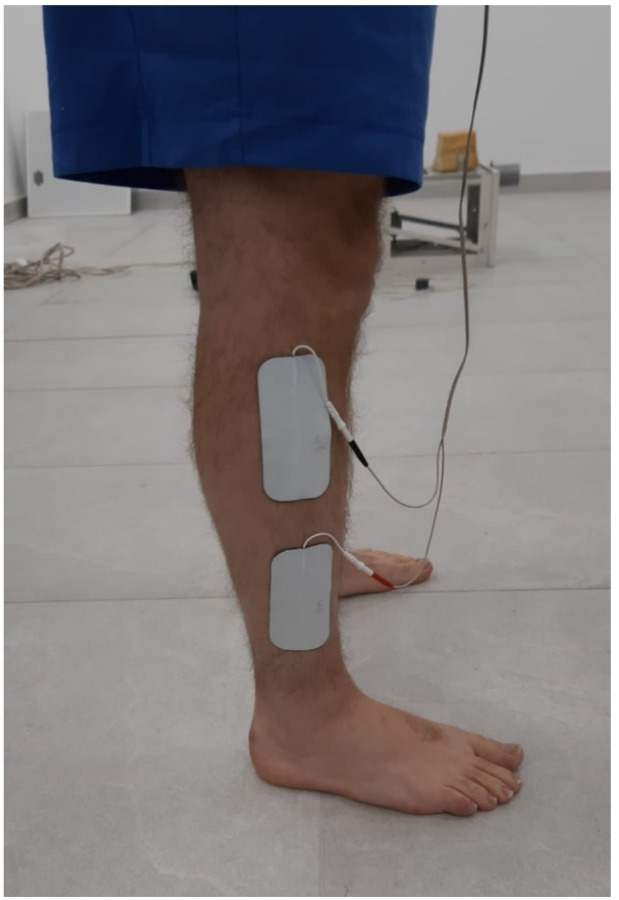
Electrodes’ placement.

Peroneal stimulation was delivered using an FES system (NESS L300Plus, Bioness, Valencia, CA, USA). The system consists of three components that communicate via radio frequency signals: (1) a stimulator, (2) a gait sensor that is placed under the heel, and (3) and control unit. An algorithm analyzes the gait sensor’s data to detect gait events (i.e., heel strike and toe-off) in real time [61]. The participants walked with shoes on the treadmill at their SSP while receiving peroneal FES for 10 min. To resemble daily activity, participants were instructed to use their mobile phones while walking. Adhesive electrodes (5 × 10 cm) were placed just below the head of the fibula and over the peroneus longus belly (Figure 1). The output pulse is a biphasic symmetrical pulse with a pulse frequency of 35 Hz and pulse width of 200 μs. Stimulation amplitude was initially set in a seated position to achieve a visible ankle eversion movement that will assist with ankle stability and reduce the level of inversion during stance. The stimulation was delivered between 0% and 80% of the stance phase. These activation times were chosen based on subjective comfort reported by participants in an early pilot study. The stimulation amplitude was readjusted during walking to make sure that it was not interfering with a normal gait. The stimulation intensity for most subjects ranged between 33 mA and 40 mA. The intervention was applied to the affected limb in the CAI group and was randomly chosen for the control group. In the case of bilateral CAI, the limb with the lower CAIT score was chosen.

### 2.6. Data Processing and Statistical Analysis

To compare between-group baseline characteristics, nonparametric Mann–Whitney U tests and *Χ^2^* tests were used for continuous and categorical variables, respectively. Time series data (frontal plane mean angles and normalized peroneal EMG) were plotted throughout the stance phase with their corresponding 95% confidence intervals (CI) [62]. A significant, meaningful difference was defined in case a nonoverlapping CI was demonstrated for consecutive 3% of the stance [62,63]. Additionally, a 2 × 2 mixed analysis of variance (ANOVA) was conducted using Statistical Parametric Mapping (SPM), to compare the between-group (group), within-group (time), and interaction (group × time) effects on the ankle’s frontal plane angle and muscle activation normalized amplitude. In the case of significant interaction, pairwise paired *t*-tests with Bonferroni correction for multiple comparisons were conducted, to assess the within-group (pre- vs. post-intervention) and between-group (CAI vs. control, pre- vs. post-intervention) differences. The significance level was set as *p* < 0.05. Python 3.8 was used for all data processing. Statistical analysis was conducted using R v.4.0.3 (R Foundation for Statistical Computing, Vienna, Austria) and SPM1d v.0.4 package for Python 3.8 [64].

## 3. Results

In total, 24 participants with CAI (7 women and 17 men, 30.5 ± 6.3 years) and 24 controls (11 women and 13 men, 30.0 ± 6.6 years) completed this study. Their demographic characteristics are presented in Table 1. There were no differences in baseline characteristics (age, gender, BMI) between groups. The average CAIT scores for the intervention leg were 14.0 ± 6.1 and 29.0 ± 1.4 in the CAI and control groups, respectively (*p* < 0.001). The average FAAM–ADL and FAAM–sports scores were 90.4 ± 10.4 and 62.7 ± 15.0 in the CAI group and 99.4 ± 1.6 and 98.1 ± 3.5 in the control group (*p* < 0.001)

### 3.1. Ankle Kinematics

**Figure 2:**
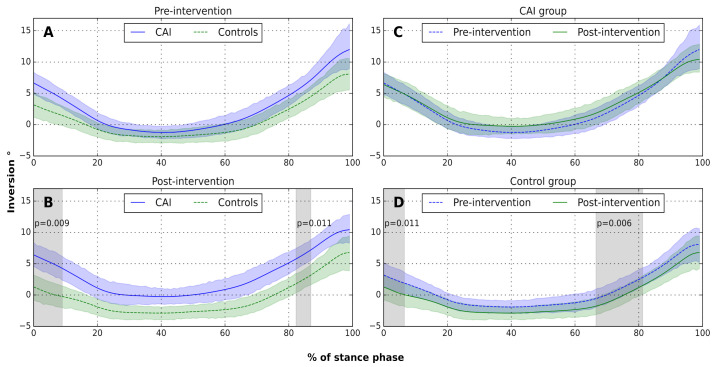
Between- and within-group comparisons of ankle inversion ankle during the stance phase of gait: (**A**) before intervention, between groups; (**B**) after intervention, between groups; (**C**) CAI group, pre/post-intervention; (**D**) control group, pre/post-intervention. Solid and dashed lines represent group means. Green and blue shaded areas represent 95% confidence intervals of the mean, at each data point. Gray shaded areas represent significant differences according to SPM analysis (*p* < 0.0125, due to Bonferroni correction).

The SPM model for ankle inversion angle demonstrated significant main effects for group between 0% and 12% (*p* = 0.024) and between 77% and 91% (*p* = 0.018) of stance, and significant group x time interaction between 39% and 82% (*p* < 0.001) of stance. No significant main effect was found for time. Post hoc between-group and within-group comparisons are displayed in Figure 2. No significant differences were demonstrated between groups before intervention. However, post-intervention, the control group had significantly more ankle eversion than the CAI group between 0% and 9% (*p* = 0.009) and between 82% and 87% (*p* = 0.011) of the stance phase. No within-group differences in ankle inversion angle were demonstrated in the CAI group. However, after the intervention, the control group demonstrated more ankle eversion than before the intervention between 0% and 7% (*p* = 0.011) and between 67% and 81% (*p* = 0.006) of the stance phase (Figure 2D).

### 3.2. Peroneal Activity

**Figure 3:**
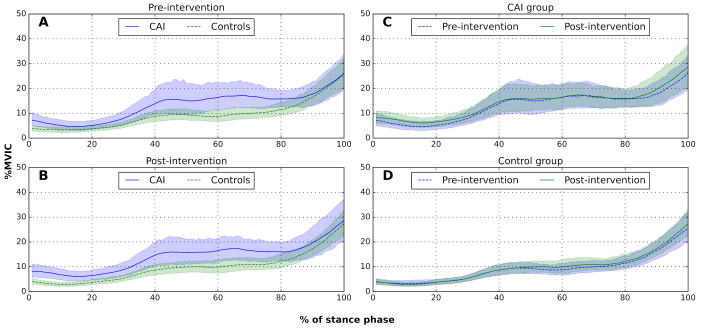
Between- and within-group comparisons of peroneal electromyography during the stance phase of gait: (**A**) before intervention, between groups; (**B**) after intervention, between groups; (**C**) CAI group, pre/post-intervention; (**D**) control group, pre/post-intervention. Solid and dashed lines represent group means. Green and blue shaded areas represent a 95% confidence interval of the mean, at each data point.

The SPM model for peroneal EMG did not yield any significant main or interactive effects. Figure 3 presents the average peroneal EMG across the stance phase for between- and within-groups comparisons, respectively. As displayed in Figure 3B, a between-group trend (nonoverlapping, 95% CI) was evident at post-intervention, with the CAI group demonstrating a higher peroneal activity than the control group.

### 3.3. Secondary Analysis

After reviewing the study results, a secondary post hoc analysis was conducted to explore a possible mechanism for the between-group difference in the kinematic adaptation to FES. We assumed that this finding might be the result of an earlier onset of peroneal fatigue in the CAI group (see the Discussion Section below). Muscle fatigue can be assessed with further signal processing of EMG data. A reduction in EMG median-frequency (MDF) and an increase in the signal root mean square (RMS) have been suggested to indicate muscle fatigue [65]. Therefore, MDF and RMS were calculated for the 200 ms immediately after heel strike for each step, and the median value per participant and measurement was used for analysis. Then, two 2 × 2 mixed ANOVAs were conducted to assess the effect of the group and the FES intervention on MDF and RMS. The secondary EMG analyses are presented in Table 2. For MDF, a significant main effect was found for the FES intervention (*F*_(1,46)_ = 16.64, *p* < 0.001) but not for group or group x intervention interaction. For RMS, a significant main effect was found for group (*F*_(1,46)_ = 0.68, *p* = 0.41), as well as significant group × intervention interaction effect (*F*_(1,46)_ = 4.3, *p* = 0.04). A trend for intervention effect was also found (*F*_(1,46)_ = 3.04, *p* = 0.08).

## 4. Discussion

The current research investigated the effect of peroneal FES on gait patterns of individuals with CAI. Specifically, we examined the immediate carryover effects of peroneal FES on ankle frontal plane kinematics and peroneal activity throughout the stance phase of gait. Contrary to our hypothesis, peroneal FES did not normalize the walking pattern of people with CAI. However, healthy controls demonstrated increased ankle eversion angle at early and late stance after the intervention. Peroneal EMG activity was not affected by the intervention in either group. To the best of our knowledge, this study is the first to report these results.

Our results show that immediate kinematic adaptation was observed in the control group in the current study but not in the CAI group. The different adaptation to FES of subjects with and without CAI may be related to muscle fatigue resulting in a decrease in power production in response to repeated contractile activity. Compared with voluntary muscle contraction, ES may induce greater muscle fatigue due to altered muscle recruitment patterns [41]. During voluntary muscle contraction, asynchronous contraction of motor units decreases prolonged muscle fatigue. ES mainly recruits the muscle fibers that are under the stimulating electrodes and does not allow for motor units rotation due to the fixed frequency. Low peroneal endurance was previously suggested to be the primary reason for excessive ankle inversion through the stance phase in individuals with CAI [66]. This is partially supported by a study that reported lower plantar- and dorsiflexor endurance in subjects with CAI, compared with healthy individuals [67]. Although we could not find any studies that investigated evertor muscle endurance in this population, individuals with CAI are probably more prone to develop evertor fatigue, compared with healthy controls. Therefore, it may be presumed that the intervention resulted in kinematic adaptation in the control group, but it did not affect the CAI group due to muscle fatigue.

The assumption that muscle fatigue influenced the different adaptation patterns between the groups was investigated in a secondary post hoc analysis. Assessing muscle fatigue using EMG signal can be performed by inspecting the time-domain features (i.e., RMS) or the frequency-domain features (i.e., MDF) [65]. Fast-twitch, high-frequency muscle fibers fatigue earlier than slow-twitch, low-frequency muscle fibers. This phenomenon is represented by the EMG MDF shifts toward lower frequency [65]. The secondary analysis of the EMG signal has found that compared with baseline, both groups demonstrated lower MDF values after the intervention. When inspecting time-domain features such as RMS, a fatigued muscle is expected to produce higher RMS without a change in force production [65]. The main finding of the secondary analysis regarding RMS was a group × measurement interaction. It appears that while the intervention did not affect the RMS values in the control group, the CAI group demonstrated an increase in RMS. It should be reminded that this increase in muscle activation was not accompanied by ankle movement, which was changed in the CAI group post the intervention. Thus, the secondary analysis supports our assumption that muscle fatigue was a possible underlying mechanism for the different adaptations between the groups.

ES-induced muscle fatigue can be reduced by manipulating several factors, such as stimulation parameters, training duration, and electrode position and size [68]. The FES session in the present study lasted for 10 min. Previous studies applied a wide variety of FES durations, ranging from less than a minute to 15 min [45,69]. It may be presumed that a shorter training duration may have minimized muscular fatigue. Furthermore, gradually increasing the stimulation intensity throughout the FES session may provide better adaptation and delay the onset of muscle fatigue. Using multiple electrodes with asynchronous activation patterns is another method to minimize muscle fatigue when applying ES. In the present study, we used relatively large (50 cm^2^) electrodes to apply the stimulation. While larger electrodes may produce less discomfort, they may recruit a larger portion of motor units, resulting in earlier fatigue onset. Future studies should address these issues, by applying shorter FES durations and decreasing electrodes’ size.

An additional factor that might have influenced the results of the present study is related to the stimulation sensation. The sensation of electrical current during FES may contribute to sensorimotor excitability and enhanced muscle contraction [70]. Burcal and Wikstrom [71] reported on impaired sensation in the lower limb in subjects with CAI, compared with healthy controls. Furthermore, reduced spinal and cortical excitability and altered sensorimotor control patterns are evident in CAI [32,72]. Thus, it may be presumed that these deficits affected the ability of individuals with CAI to adapt their gait patterns following FES.

Individuals with CAI demonstrate a more inverted ankle position at early stance, which may put them at greater risk for re-injury [25,56]. An intervention that will reduce the inversion angle in this population may reduce the risk for re-injury. The control group demonstrated a more everted ankle position during early (0–12%) and toward the final (77–91%) stance phase following the FES intervention. Therefore, it seems that the intervention has the potential to achieve the desired adaptation. A modified protocol (e.g., gradual training volume) may be beneficial for people in CAI. It is recommended that future studies will evaluate the efficacy of varied FES protocols to enhance gait kinematic in subjects with CAI.

Several studies have investigated EMG patterns in individuals with CAI and reported increased peroneal muscle activation during the stance phase of gait, compared with healthy controls [28,29,30]. Several explanations have been proposed as the underlying mechanism for this altered peroneal activation pattern, such as impaired feed-forward motor control, inability to produce effective muscle contraction due to reduced spinal excitability, or early fatigue [28,32]. In our study, EMG activity did not differ between subjects with or without CAI. The only near-significant finding regarding EMG activation was observed in the between-group comparison of peroneal activity post-intervention (Figure 3B). The nonoverlapping CIs suggested that following FES the CAI group had a trend for increased peroneal activation, compared with the control group. Identifying differences in time-series data by looking at the nonoverlapping areas of CIs was previously used to demonstrate between-group differences in EMG activity [62,63], but it should be acknowledged that this approach is less robust than the SPM analysis. The near-significant difference, which was observed post- but not pre-intervention, may further support the assumption of fatigue in the CAI group, as the increased muscle activation did not affect ankle position.

The findings of this study should be interpreted with some limitations. Our control group included five subjects who had a history of an ankle sprain. It may be argued that these individuals could be referred to as ankle sprain copers, not healthy controls. However, we do not believe that the inclusion of these subjects had a major effect on the results, as all ankle sprains occurred long before participating in the study, and all subjects reported no activity limitation due to their ankle history. The question of how long an individual should be considered as an ankle sprain coper after an injury is of interest for CAI studies and should be discussed in future studies. Furthermore, in this study, we only examined the immediate carryover effects of FES. This type of research is not suitable to draw clinical conclusions, but it may suggest a different adaptational pattern to FES in individuals with CAI. Several confounding variables may have influenced our results. For example, direct measurement of peroneal muscle strength and endurance could have assisted in accepting or refuting the presumption that muscle fatigue is the differing factor between the groups. Ankle characteristics such as passive instability tests and passive range of motion measurements could also better describe the study’s sample. Future studies should try to control for these factors by directly measuring fatigue and neural excitability before and after the FES intervention. Different intervention protocols should also be used to better understand the different adaptation patterns between individuals with and without CAI. Studies that will evaluate the therapeutic effect of several training sessions are also warranted.

To the best of our knowledge, this is the first study that investigated the immediate carryover ankle kinematics effects of FES in individuals with CAI. The findings of this study, which demonstrate ankle kinematics modifications in healthy controls, should encourage future research to understand how to successfully transfer these findings to people with CAI.

## Figures and Tables

**Table 1:** Demographic characteristics of the participants.

	CAI (*n* = 24)		Controls (*n* = 24)		*U*	*p*
	Mean (SD)	Median (IQR)	Mean (SD)	Median (IQR)		
Age	30.5 (6.3)	30.0 (8.8)	30.0 (6.6)	28.0 (8.0)	307	0.702
Stature (m)	1.74 (0.1)	1.74 (0.1)	1.71 (0.1)	1.7 (0.2)	340	0.287
Body mass (kg)	76.4 (12.6)	78.0 (18.8)	70.7 (12.4)	72.5 (18.8)	359	0.146
BMI (kg/m^2^)	25.1 (3.6)	25.2 (5.1)	24.1 (3.1)	23.5 (4.3)	338	0.307
CAIT (intervention)	14.0 (6.1)	15.5 (10.2)	29.0 (1.4)	30.0 (2.2)	0	<0.001
CAIT (control)	21.2 (7.0)	22.5 (10.0)	29.2 (1.2)	30.0 (2.0)	84.5	<0.001
FAAM (ADL)	90.4 (10.4)	94.0 (14.1)	99.4 (1.6)	100.0 (0.0)	64	<0.001
FAAM (sport)	62.7 (15.0)	67.9 (19.6)	98.1 (3.5)	100.0 (3.6)	0	<0.001
SSP + 20% (m/s)	1.6 (0.19)	1.6 (0.22)	1.5 (0.2)	1.5 (0.29)	342.5	0.264
	***n***	**%**	***n***	**%**	***X^2^*_(1)_**	***p***
Gender (women)	7	29.1%	11	45.8%	0.8	0.371
Previously sprained an ankle	24	100%	5	20.80%	28.2	<0.001
Intervention leg (Right)	15	62.5%	13	54.2%	0.08	0.769
Bilateral CAI	15	62.5%				

CAI: chronic ankle instability; BMI: body mass index; CAIT: Cumberland Ankle Instability Tool; FAAM: Foot and Ankle Ability Measure; ADL: activities of daily living; SSP: self-selected pace.

**Table 2:** Secondary analysis of EMG signal at 200 ms after heel strike.

		CAI		Controls		Between Groups	Between Measurements	Group × Measurement Interaction
		Mean (SD)	Median (IQR)	Mean (SD)	Median (IQR)			
MDF (Hz)	Pre	101.9 (30.8)	92.3 (58.7)	105.9 (33.9)	109.1 (50.3)	*F*_(1,46)_ = 0.68, *p* = 0.41	*F*_(1,46)_ = 16.64, *p* < 0.001	*F*_(1,46)_ = 0.07, *p* = 0.79
	Post	90.3 (35.7)	100.7 (42.0)	94.2 (32.2)	92.3 (50.3)			
RMS (%MVIC × 10^3^)	Pre	7.3 (7.6)	5.3 (5.1)	4.6 (3.1)	3.9 (4.7)	*F*_(1,46)_ = 4.53, *p* = 0.04	*F*_(1,46)_ = 3.04, *p* = 0.08	*F*_(1,46)_ = 4.3, *p* = 0.04
	Post	8.4 (7.2)	6.5 (7.7)	4.6 (3.1)	3.7 (4.4)			

CAI: chronic ankle instability; Pre: pre-intervention; Post: post-intervention; MDF: median frequency; RMS: root mean square; %MVIC: percent of maximal voluntary isometric contraction.

## Data Availability

The datasets used and/or analyzed during the current study are available from the corresponding author on reasonable request.
